# Chromobox Homologue 7 Acts as a Tumor Suppressor in Both Lung Adenocarcinoma and Lung Squamous Cell Carcinoma via Inhibiting ERK/MAPK Signaling Pathway

**DOI:** 10.1155/2022/4952185

**Published:** 2022-05-19

**Authors:** Jinlong Huang, Weiqing Zhang, Dongliang Lin, Luoyu Lian, Wenshan Hong, Zhendong Xu

**Affiliations:** Department of Thoracic Surgery, Quanzhou First Hospital, Quanzhou, China

## Abstract

Chromobox homologue 7 (CBX7) is a member of the polycomb group family that plays a pivotal role in regulating cellular processes in human cancers. This study aims to explore the function and underlying molecular mechanisms of CBX7 in lung adenocarcinoma (LUAD) and lung squamous cell carcinoma (LUSC). The expression of CBX7 in LUAD and LUSC tissues was analyzed by UALCAN and GEPIA based on the TCGA database. Cell viability and apoptosis were measured by CCK-8 and flow cytometry assays, respectively. Cell migration and invasion were detected by transwell assay. The functions of downregulated genes in LUAD were enriched via GO and KEGG pathway analyses. The mRNA expression of CBX7, ERK1/2, and p38 was determined by qRT-PCR, and the protein levels of CBX7, ERK1/2, p-ERK1/2, p38, and p-p38 were measured by Western blotting. Tumor xenograft model was established to validate the antitumor effect of CBX7. The expression of CBX7 and Ki-67 in tumor tissues was detected by immunohistochemistry. CBX7 was downregulated in the tissues and cells of both LUAD and LUSC. Low CBX7 expression was associated with a poor overall survival rate in LUAD patients. CBX7 overexpression inhibited the viability, migration, and invasion and promoted the apoptosis of LUAD and LUSC cells. In addition, the downregulated genes in LUAD were enriched in MAPK cascade (GO) and MAPK signaling pathway (KEGG). ERK/MAPK pathway was then determined as a downstream target of CBX7, which was inhibited by CBX7 overexpression in LUAD and LUSC cells. The overexpression of CBX7 inhibited the malignant progression of LUAD and LUSC cells probably via suppressing the ERK/MAPK signaling pathway *in vitro* and *in vivo*.

## 1. Introduction

Lung cancer is one of the leading causes of malignancy-related mortality worldwide [1]. As the most common type of lung cancer, nonsmall cell lung cancer (NSCLC) accounts for 85% of lung cancer. According to the heterogeneous background of NSCLC, lung adenocarcinoma (LUAD) and lung squamous cell carcinoma (LUSC) are two typical subtypes of NSCLC [2, 3]. Until now, despite remarkable progress in NSCLC treatment, the prognosis of NSCLC patients is still unsatisfactory, along with no prominent reduction in mortality [4]. Therefore, it is urgently needed to find effective molecular targets and identify related molecular mechanisms for the treatment of NSCLC.

Chromobox homolog 7 (CBX7) is a member of the polycomb group family, which acts as a tumor suppressor in multiple human cancers, including ovarian carcinoma, pancreatic cancer, and cervical cancer [5–8]. Ni et al. [7] found that CBX7 is downregulated in pancreatic ductal adenocarcinoma, and it can suppress cell proliferation, migration, and invasion. Li et al. [8] indicated that CBX7 exerts a suppressive effect on the growth and motility of cervical cancer cells. In addition, a study on human lung carcinomas showed that CBX7 restoration increases the susceptibility of irinotecan treatment for tumor cells [9]. However, the function and underlying mechanisms of CBX7 in NSCLC have not been fully revealed.

ERK/MAPK signaling pathway plays a pivotal role in regulating cell growth, differentiation, and migration, which is closely associated with NSCLC development and progression [10]. Liu et al. [11] revealed that MALAT1 promotes the growth, migration, and invasion of NSCLC cells via activating the ERK/MAPK pathway. Zhang et al. [12] disclosed that the transcription factor c-MYC activates ERK/MAPK pathway to drive the development and progression of NSCLC. In addition, a previous study also indicated that miR-18a targeting CBX7 exerts suppressive effects on ovarian carcinoma cells via modulating ERK/MAPK pathway [5]. However, it remains unclear whether CBX7 is involved in the progression of NSCLC via regulating the ERK/MAPK signaling pathway.

In this research, the expression of CBX7 in LUAD and LUSC was firstly evaluated by bioinformatics analysis, and its function was then validated in vitro. Furthermore, the underlying action mechanism of CBX7 involving the ERK/MAPK pathway was uncovered in LUAD and LUSC cells. This study may provide an effective therapeutic target for NSCLC and offer the underlying theoretical foundation for NSCLC treatment.

## 2. Methods

### 2.1. Data Collection and Bioinformatics Analyses

The RNA-seq data and corresponding clinical information of LUAD and LUSC samples were collected from The Cancer Genome Atlas (TCGA) (https://cancergenome.nih.gov/). Differential expression genes (DEGs) in LUAD were screened with the thresholds of adjusted *p* value < 0.05 and |log_2_FC| > 1 by the *limma* package in R [13]. A volcano plot including DEGs was obtained using the *ggplot2* package of R software [14]. The CBX7 expression in LUAD and LUSC samples and normal samples was analyzed by UALCAN (https://ualcan.path.uab.edu/analysis.html) based on the TCGA database. The associations between CBX7 expression and overall survival (OS) of LUAD patients were also analyzed using the Gene Expression Profiling Interactive Analysis (GEPIA) (https://gepia.cancer-pku.cn/).

### 2.2. Cell Culture

Human pulmonary alveolar epithelial cell line (HPAEpiC), LUAD cell line (A549), and LUSC cell line (SK-MES-1) were purchased from the American Type Culture Collection (ATCC; MD, USA). HPAEpiC and A549 cells were cultured in Roswell Park Memorial Institute (RPMI; Gibco, NY, USA). 1640 supplemented with 10% fetal bovine serum (FBS; Gibco, NY, USA) and 1% penicillin/streptomycin. SK-MES-1 cells were cultured in Dulbecco's modified Eagle's medium (DMEM; Gibco, NY, USA) containing 10% FBS and 1% penicillin/streptomycin. All cell lines were maintained at 37°C with 5% CO_2_.

### 2.3. Cell Transfection

The overexpression of CBX7 in A549 and SK-MES-1 cells was constructed using a lentivirus packaging system. CBX7 (lenti-CBX7) and negative control (lenti-NC) were packaged in lentivirus. A549 and SK-MES-1 cells were trypsinized and diluted to 5 × 10^5^ cells/mL and then plated in six-well cell culture plates (100 *µ*L/well) for culturing at 37°C with 5% CO_2_. When cell confluence reached 80%, 200 *µ*L of lentivirus solutions (lenti-NC and lenti-CBX7 with titration of 1 × 10^8^ TU/mL) were added to A549 and SK-MES-1 cells for transfection. Cells were incubated with the lentivirus solutions for 72 h. The untransfected cells were used as the control.

### 2.4. Cell Viability Assay

The viability of transfected A549 and SK-MES-1 cells was assessed using the Cell Counting Kit-8 (CCK-8) assay. Cell suspensions (5 × 10^5^ cells/mL) were seeded into 96-well plates (100 *μ*L/well) for incubating at 37°C with 5% CO_2_. Then, cells cultured for 24 h, 48 h, and 72 h were, respectively, incubated with 10 *μ*L CCK-8 reagent (Beyotime, China) for 2 h. The absorbance was measured under a wavelength of 450 nm using a DR-200 Bs microplate reader (Diatek, China).

### 2.5. Flow Cytometry

The apoptosis of A549 and SK-MES-1 cells was assessed using the Annexin V-FITC apoptosis kit (Beyotime, China) according to the manufacturer's instructions. In brief, 300 *μ*L resuspended cells (5 × 10^5^ cells/mL) were incubated with 5 *μ*L Annexin V-FITC for 15 min and then with 10 *μ*L propidium iodide (PI) for 10 min at room temperature in the dark. Afterwards, the apoptosis rate was detected using a flow cytometry (Beckman Coulter, Germany) with CellQuest software (BD Biosciences, NJ, USA).

### 2.6. Transwell Assay

The migration and invasion ability of A549 and SK-MES-1 cells were evaluated using a transwell assay. For migration assay, 200 *μ*L cell suspensions (1 × 10^5^ cells/mL) were added to the upper chamber, and 600 *μ*L media containing 10% FBS were added to the lower chamber. After incubated for 24 h, cells on the lower chamber were fixed in methanol for 30 min and then stained with crystal violet for 20 min. For invasion assay, cells were treated with the above procedures after covered Matrigel (BD Biosciences, CA, USA) on the upper chamber of the transwell. The cell images were photographed by an optical microscope (20× magnification, Olympus, Japan) by randomly selecting five fields for each group.

### 2.7. Functional Enrichment Analysis

The functions of downregulated genes in LUAD were enriched by the Gene Ontology (GO) and Kyoto Encyclopedia of Genes and Genomes (KEGG) analyses using Metascape (https://metascape.org/). Relevant parameters were set as follows: minimum overlap = 3, *p* value cutoff = 0.01, and minimum enrichment = 1.5.

### 2.8. Quantitative Real-Time PCR (qRT-PCR)

Total RNA was extracted from HPAEpiC, A549, and SK-MES-1 cells using TRIzol reagent (Invitrogen, CA, USA) and reversely transcribed to cDNA using Reverse Transcription System Kit (TransGen, China). qRT-PCR was performed using an SYBR Green PCR Master Mix (Lifeint, China) on Agilent Mx3000P QPCR System (Agilent, CA, USA). The reaction program was 95°C for 3 min and 40 cycles of 95°C for 12 s and 62°C for 40 s. The primers were designed as follows: CBX7 (forward, 5'-TGA AGT GGA AAG GAT GGC CC-3′; reverse, 5′-TCC TCC TTC TCC TCG TAG GC-3′), ERK1/2 (forward, 5′-TCA AGC CTT CCA ACC TC-3′; reverse, 5′-GCA GCC CAC AGA CCA AA-3′), p38 (forward, 5′-AGG GCG ATG TGA CGT TT-3′; reverse, 5′-CTG GCA GGG TGA AGT TGG-3′), and GAPDH (forward, 5′-ACA ACT TTG GTA TCG TGG AAG G-3′; reverse, 5′-GCC ATC ACG CCA CAG TTT C-3′). The relative mRNA expression was calculated using the 2^∆∆Ct^ method. GAPDH was used as an internal control.

### 2.9. Tumor Xenograft Model Establishment

Male BALB/c nude mice (5-week-old) were purchased from Wei-tongli-hua Laboratory Animal Technology Company (Beijing, China). Mice were maintained at 23 ± 1°C under 12 h light/12 h dark cycle and provided with sufficient food and water. Cell suspensions (1 × 10^6^ cells/mL) of A549 cells transfected with lenti-NC or lenti-CBX7 were subcutaneously injected into nude mice (*n* = 6 per group). Tumor volume (mm^3^) was measured every three days for 21 days, which was calculated as follows: tumor volume (mm^3^) = 0.5 × length × width^2^. At the end point of the experiment, tumors were harvested, photographed, and weighed. All animal experiments were conducted under the approval of the Animal Ethical and Welfare Committee of the Quanzhou First Hospital.

### 2.10. Immunohistochemistry

Paraffin-embedded tumor tissues were cut into 4 *µ*m sections that were deparaffinized, rehydrated through graded alcohol, subjected to antigen retrieval, and blocked the endogenous peroxidases. Subsequently, sections were incubated with CBX7 or Ki-67 antibody (1 : 100; Abcam, UK) at 4°C overnight, followed by incubation with secondary antibody (1 : 500) for 1 h. After washing with PBS twice, sections were stained with 0.03% 3, 3-diaminobenzidine in 0.05 M Tris-HCl (pH 7.6). The stained sections were observed under a microscope (Olympus, Japan).

### 2.11. Western Blot

Cells or tumor tissues were lysed with RIPA buffer (Takara Bio, Japan) for extracting total protein. Protein concentration was quantified using BCA Protein Assay Kit (Thermo Fisher Scientific, CA, USA). Proteins were separated using 10% SDS-PAGE and then transferred onto polyvinylidene difluoride (PVDF) membranes. Next, membranes were incubated with blocking buffer for 1 h, followed by the incubation with primary antibody against CBX7, ERK1/2, p-ERK1/2, p38, p-p38, and GAPDH (1 : 1,000, Abcam, UK) at 4°C overnight. After being washed with 1× TBST buffer for three times, membranes were incubated with horseradish peroxidase (HRP)-conjugated secondary antibody (1 : 500, MultiSciences, China) for 2 h at room temperature in the dark. The protein bands were visualized using an ECL reagent kit (Thermo Fisher Scientific, CA, USA) and observed under a ChemiDoc™ imaging system (Bio-Rad, CA, USA). The relative protein expression was calculated via normalizing to GAPDH.

### 2.12. Statistical Analysis

All data were expressed as mean ± standard deviation (SD). Differences between groups were analyzed via one-way analyses of variance (ANOVA), followed by Tukey's test. Statistical analysis was carried out using SPSS 27.0 (IBM, IL, USA). *p* < 0.05 was considered as statistically significant.

## 3. Results

### 3.1. CBX7 Expression Was Downregulated in LUAD and LUSC Tissues and Cells

**Figure 1:**
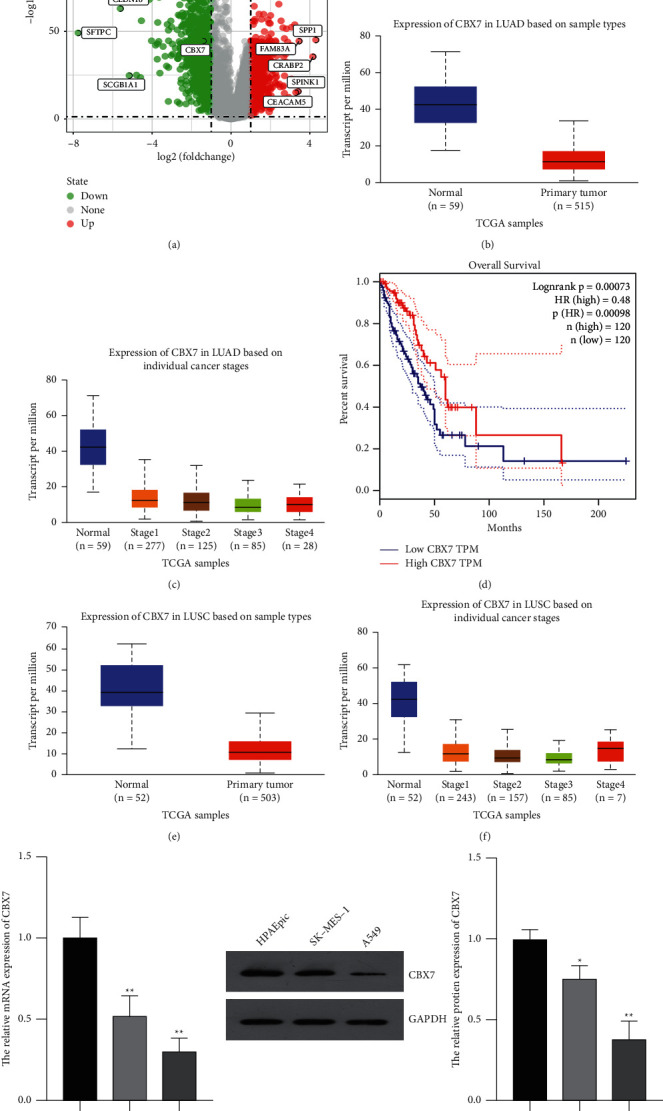
Chromobox homologue 7 (CBX7) was downregulated in lung adenocarcinoma (LUAD) and lung squamous cell carcinoma (LUSC). (a) Differentially expressed genes (DEGs) in LUAD were show in a volcano plot based on The Cancer Genome Atlas (TCGA) database (https://www.cancer.gov/). (b) CBX7 expression in LUAD tissues and normal tissues was analyzed by the UALCAN (http://ualcan.path.uab.edu/analysis.html). (c) CBX7 expression in LUAD tissues of different clinicopathological stages was analyzed by the UALCAN. (d) Overall survival of LUAD patients with low and high CBX7 expression was analyzed by GEPIA (http://gepia.cancer-pku.cn/). (e) CBX7 expression in LUSC tissues and normal tissues was analyzed by the UALCAN. (f) CBX7 expression in 6 tissues of different clinicopathological stages was analyzed by the UALCAN. (g) Relative mRNA expression of CBX7 in HPAEpiC, SK-MES-1, and A549 cells was measured by qRT-PCR. (h) Relative protein level of CBX7 in HPAEpiC, SK-MES-1, and A549 cells was measured by Western blotting. ^*∗*^*p* < 0.05 and ^*∗∗*^*p* < 0.01 vs. HPAEpiC.

The DEGs in LUAD tissues were presented as a volcano plot according to the TCGA database. As a result, we obtained 1677 DEGs including 700 upregulated and 977 downregulated genes. CBX7 was one of the downregulated genes in LUAD tissues (Figure 1(a)). Based on the UALCAN, CBX7 expression was significantly lower in LUAD tissues compared with that in normal tissues (*p*=1.62*E* − 12, Figure 1(b)). CBX7 expression in LUAD tissues also presented a decreasing trend from clinicopathological stage 1 to 4 (Figure 1(c)). Based on GEPIA, the OS of LUAD patients with low CBX7 expression was dramatically worse than those with high CBX7 expression (*p*=0.00098, Figure 1(d)). In addition, the expression of CBX7 was also decreased in LUSC tissues with progressive clinicopathological stages (Figures 1(e) and 1(f)). Furthermore, *in vitro* experiments showed that both the mRNA and protein expression of CBX7 were remarkably lower in LUAD cells (A549) and LUSC cells (SK-MES-1) than that in normal HPAEpiC cells (*p* < 0.05, Figures 1(g) and 1(h)).

### 3.2. CBX7 Overexpression Inhibited the Malignant Characteristics of LUAD and LUSC Cells

**Figure 2:**
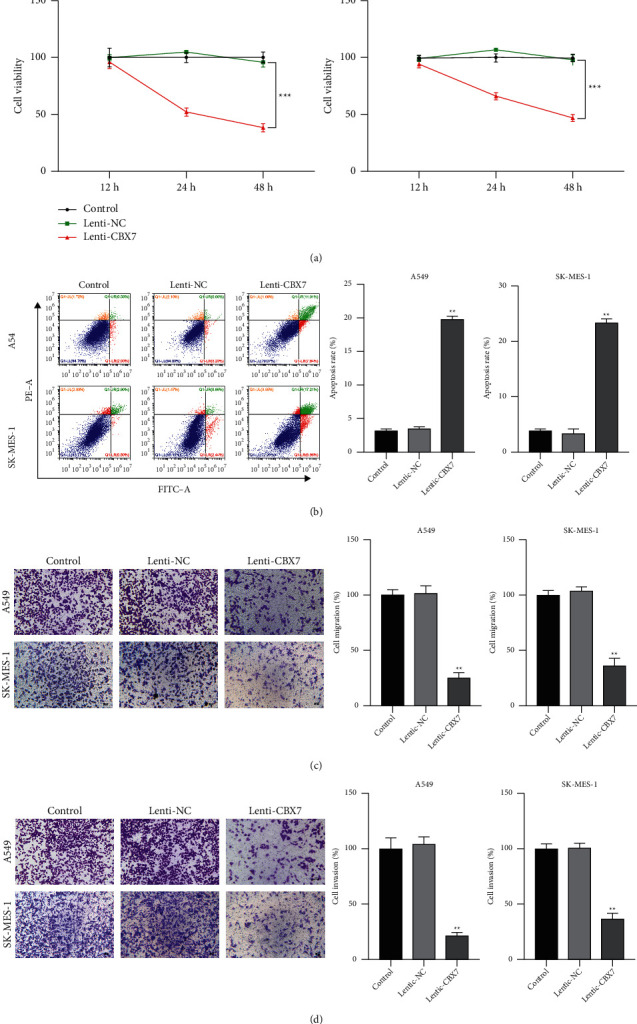
CBX7 overexpression inhibited the malignant progression of LUAD and LUSC cells. (a) The viability of A549 and SK-MES-1 cells was detected by CCK-8 assay. (b) The apoptosis of A549 and SK-MES-1 cells was detected by flow cytometry. (c) The migration of A549 and SK-MES-1 cells was measured by transwell assay. Scale bar = 50 *µ*m. (d) The invasion of A549 and SK-MES-1 cells was measured by transwell assay. Scale bar = 50 *µ*m. A549 and SK-MES-1 cells were transfected with lenti-NC or lenti-CBX7. ^*∗∗*^*p* < 0.01 and ^*∗∗∗*^*p* < 0.001 vs. lenti-NC.

The function of CBX7 in NSCLC was further identified in A549 and SK-MES-1 cells. CCK-8 assay showed that CBX7 overexpression significantly decreased the viability of A549 and SK-MES-1 cells compared with the lenti-NC (*p* < 0.01) (Figure 2(a)). In contrast, the apoptosis rate of A549 and SK-MES-1 cells presented an increasing trend after overexpression of CBX7 (*p* < 0.01) (Figure 2(b)). Moreover, the migration and invasion ability of A549 and SK-MES-1 cells were dramatically declined after CBX7 overexpression in comparison to the lenti-NC (*p* < 0.01) (Figures 2(c) and 2(d)).

### 3.3. GO and KEGG Enrichment Analyses of Downregulated Genes in LUAD

The potential functions of downregulated genes in LUAD were analyzed by GO and KEGG enrichment analyses. The results showed that the gene clusters were involved in some cancer-related GO terms, including extracellular matrix, regulation of cell adhesion, response to growth factor, and negative regulation of cell population proliferation (Supplementary Figure 1(a)). In addition, several cancer-related signaling pathways were enriched based on the KEGG database, including the TGF-beta, PPAR, Rap1, cAMP, P13K-Akt, and Jak-STAT signaling pathways (Supplementary Figure 1(b)). It is noteworthy that the MAPK cascade (GO) and MAPK signaling pathway (KEGG) were both enriched by downregulated genes in LUAD.

### 3.4. CBX7 Inhibited the ERK/MAPK Signaling Pathway in LUAD and LUSC Cells

**Figure 3:**
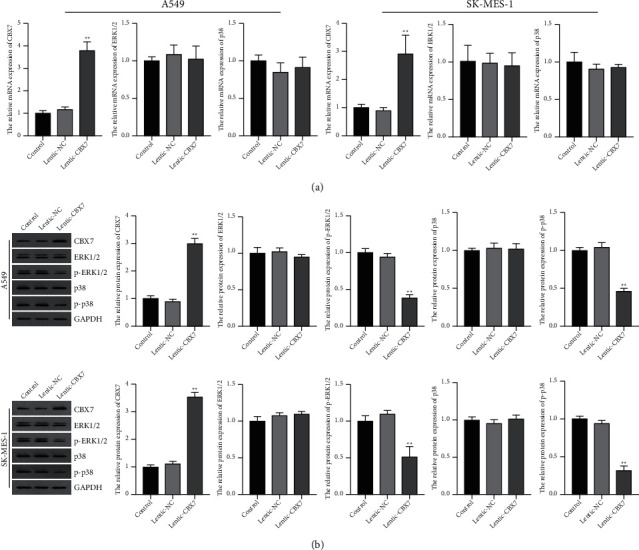
CBX7 overexpression suppressed the ERK/MAPK signaling pathway in LUAD and LUSC cells. (a) Relative mRNA expression of CBX7, ERK1/2, and p38 in A549 and SK-MES-1 cells was detected by qRT-PCR. (b) Relative protein levels of CBX7, ERK1/2, p-ERK1/2, p38, and p-p38 in A549 and SK-MES-1 cells were detected by Western blotting. A549 and SK-MES-1 cells were transfected with lenti-NC or lenti-CBX7. ^*∗∗*^*p* < 0.01 vs. lenti-NC.

Function enrichment analysis showed that ERK/MAPK signaling pathway may be an underlying mechanism of CBX7 against NSCLC, which was further validated by *in vitro* experiments. As given in Figures 3(a) and 3(b), both the mRNA and protein levels of ERK1/2 and p38 in A549 and SK-MES-1 cells were not significantly affected by CBX7 overexpression. It is noteworthy that CBX7 overexpression dramatically downregulated the protein levels of phosphorylated ERK1/2 and p38 (p-ERK1/2 and p-p38) in A549 and SK-MES-1 cells in comparison to the lenti-NC (*p* < 0.01) (Figure 3(b)).

### 3.5. CBX7 Restrained the Tumor Growth of Lung Cancer Probably via Deactivating the ERK/MAPK Pathway *In Vivo*

**Figure 4:**
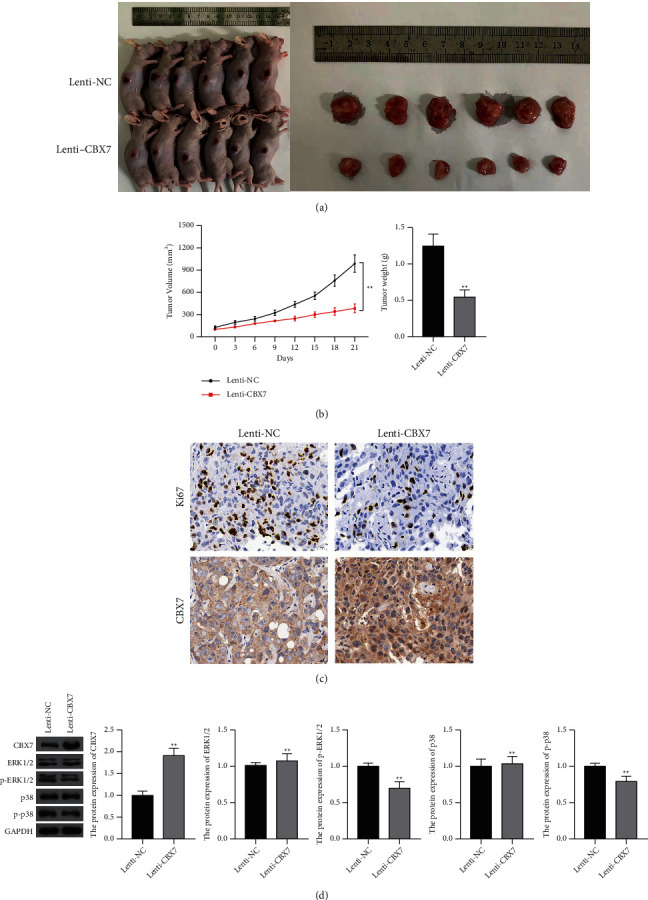
CBX7 overexpression inhibited the tumor growth of lung cancer via deactivating the ERK/MAPK pathway in vivo. (a) Representative images of the tumors generated by subcutaneously injection with A549 cells transfected with lenti-NC or lenti-CBX7. (b) Tumor volume and tumor weight. (c) The expression of CBX7 and Ki-67 in tumor tissues was determined by immunohistochemistry. Scale bar = 20 *µ*m. (d) The protein levels of CBX7, ERK1/2, p-ERK1/2, p38, and p-p38 in tumor tissues were measured by Western blotting. ^*∗∗*^*p* < 0.01 vs. lenti-NC.

To further confirm and evaluate the role of CBX7 in LUAD and LUSC *in vivo*, tumor xenograft model was established by subcutaneous injection with A549 cells transfected with lenti-NC or lenti-CBX7. Consistent with the *in vitro* study, the overexpression of CBX7 suppressed the tumor growth in mice compared with the lenti-NC (Figure 4(a)). Tumors were lighter and grew slower in the CBX7 overexpression group than those generated by the lenti-NC (*p* < 0.01) (Figure 4(b)). In addition, immunohistochemistry showed the increased CBX7 and the decreased Ki-67 (a cell proliferation biomarker) expression in the lenti-CBX7 group compared with that in the lenti-NC group (Figure 4(c)). Furthermore, the mechanism of CBX7 suppressing LUAD and LUSC involving in the ERK/MAPK pathway was verified *in vivo*. Western blotting showed that CBX7 overexpression significantly restrained the protein expression of p-ERK1/2 and p-p38 in tumor tissues in comparison to the lenti-NC (*p* < 0.01) (Figure 4(d)).

## 4. Discussion

LUAD and LUSC are the major histological types of NSCLC with poor prognosis and high mortality globally [15]. Recently, CBX7 has been reported to play a crucial role in the processes of multiple cancers [7, 8, 16]. In the present study, we found that CBX7 expression was downregulated in LUAD and LUSC tissues and cell lines. CBX7 overexpression inhibited the malignant progression of LUAD and LUSC cells, and this process was closely associated with the ERK/MAPK signaling pathway.

CBX7 presents a downregulated expression in multiple cancers, including pancreatic, cervical, breast, and colon cancers [7, 8, 17–19]. The downregulation of CBX7 is closely correlated with cancer aggressiveness and poor prognosis [6]. For instance, loss of CBX7 expression represents an adverse prognostic marker for the survival of patients with colon cancer [19]. Decreased CBX7 expression is an independent predictor of the poor prognosis of cervical cancer [18]. According to the TCGA database, we found that CBX7 was also downregulated in LUAD and LUSC tissues and was negatively associated with advanced clinical stages. Moreover, the OS of LUAD patients with low CBX7 expression was worse than the patients with high CBX7 expression. These results indicate that the low CBX7 expression was related to the poor prognosis of NSCLC, which are consistent with previous studies in cervical and colon cancers [18, 19]. Furthermore, considerable evidence has verified that CBX7 possesses an antitumor effect in cancers. Huang et al. [16] indicated that CBX7 overexpression inhibits the proliferation, migration, invasion, and cancer stemness of urinary bladder cancer cells. Li et al. [8] also revealed that CBX7 suppresses the proliferation and induces the apoptosis of cervical cancer cells. We speculated that CBX7 may also serve as a tumor suppressor in LUAD and LUSC. To verify this hypothesis, a series of functional experiments *in vitro* were performed. Our results showed that CBX7 overexpression inhibited the viability, migration, and invasion and promoted the apoptosis of LUAD and LUSC cells. In vivo experiments also showed that CBX7 overexpression suppressed tumor growth and Ki-67 (a cell proliferation biomarker) expression. These findings prove that CBX7 is a tumor suppressor in inhibiting the malignant progression of LUAD and LUSC cells.

To explore the underlying molecular mechanisms of CBX7 against NSCLC, the biological functions of all downregulated genes in LUAD were enriched by GO and KEGG analyses. The results showed that the MAPK may be a potential action mechanism, evidenced by its enrichment in both GO and KEGG terms. MAPK cascade is a highly conserved module involved in the regulation of various cellular processes in cancers, including cell proliferation, differentiation, and migration [20]. The extracellular signal-regulated kinases ERK1 and ERK2 are conserved and ubiquitous serine-threonine kinases involved in ERK/MAPK signaling pathway, which also play critical roles in cancer development and progression [10]. Until now, many genes have been reported to be involved in the progression of NSCLC by regulating ERK/MAPK pathway, such as c-MYC [12], MALAT1 [11], HMGB3 [21], and FAK [22]. In addition, a previous study suggested that CBX7 can suppress the progression of ovarian carcinoma by inhibiting the ERK/MAPK pathway [5]. Our study found that CBX7 overexpression reduced the protein levels of p-ERK1/2 and p-p38 in LUAD and LUSC cells and tumor tissues. This result demonstrates that CBX7 inhibits the ERK/MAPK signaling pathway. To combine with the antitumor effect of CBX7, we suspect that CBX7 may suppress the malignant progression of NSCLC probably by inhibiting the ERK/MAPK signaling pathway.

In summary, CBX7 is downregulated in LUAD and LUSC, which may be a biomarker for the poor prognosis. CBX7 suppressed the malignant progression of LUAD and LUSC probably via inhibiting the ERK/MAPK signaling pathway *in vitro* and *in vivo*. These findings provide a potential prognostic biomarker and therapeutic target for NSCLC. However, our study is still lack of *in vivo* experiments to verify our findings. The underlying mechanism of CBX7 involving the ERK/MAPK pathway is also needed to be further elucidated.

## Data Availability

The datasets used and analyzed during the current study are available from the corresponding author on reasonable request.

## Supplementary Materials

Supplementary Materials Supplementary Figure 1: functional enrichment analyses of downregulated genes in LUAD tissues. (A) Gene Ontology (GO) enrichment analysis. (B) Kyoto Encyclopedia of Genes and Genomes (KEGG) pathway analysis. Both GO and KEGG analyses were performed on the Metascape server (https://metascape.org/) with the thresholds of minimum overlap = 3, *p* value cutoff = 0.01, and minimum enrichment = 1.5.
