# Supplementary figures and images for: Chromobox Homologue 7 Acts as a Tumor Suppressor in Both Lung Adenocarcinoma and Lung Squamous Cell Carcinoma via Inhibiting ERK/MAPK Signaling Pathway
Source: Evid Based Complement Alternat Med. 2022 May 19;2022:4952185. doi: 10.1155/2022/4952185 (PMC9135519; doi:10.1155/2022/4952185)

A

## GO enrichment

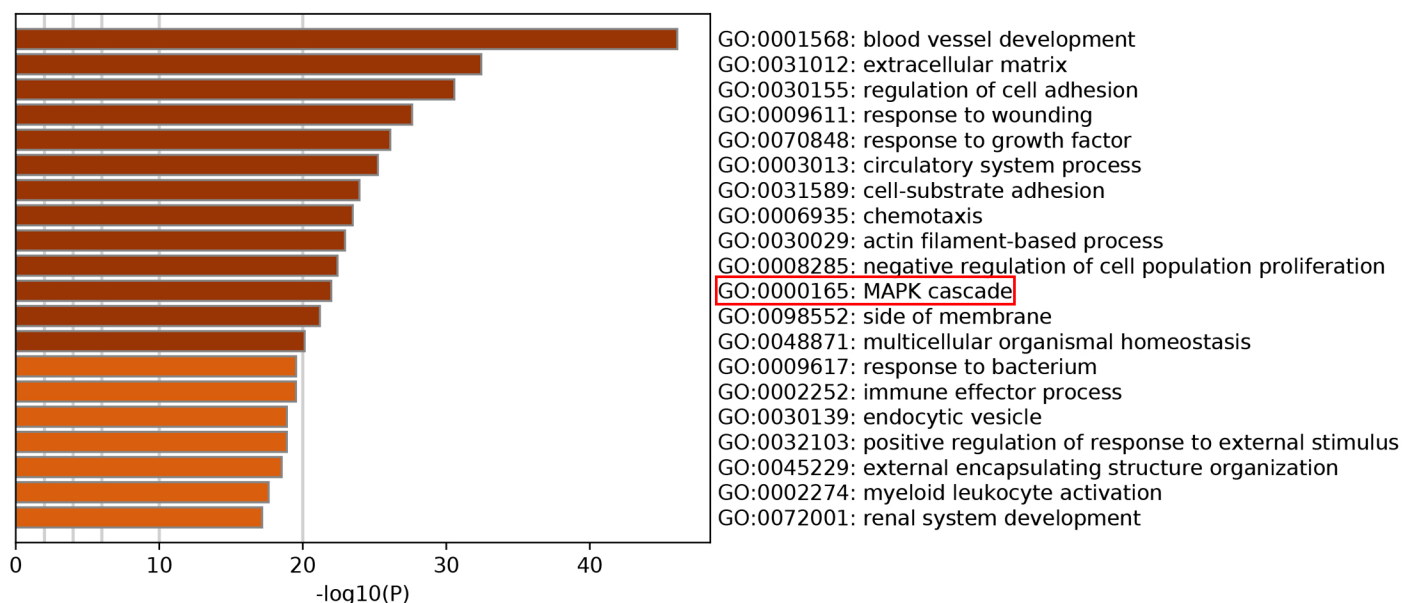

B

## KEGG enrichment

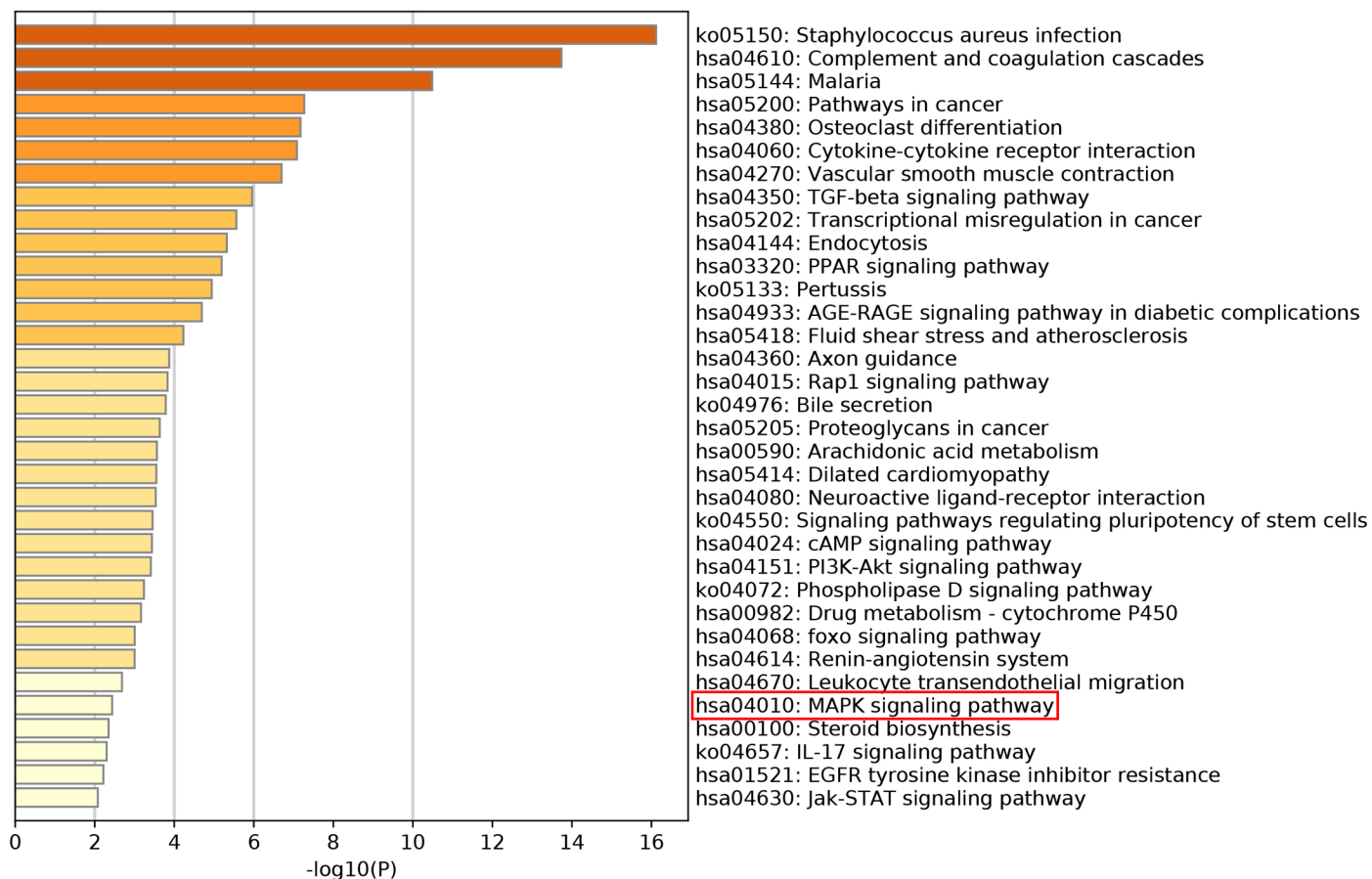

Supplement: Supplementary Materials — Supplementary Figure 1: functional enrichment analyses of downregulated genes in LUAD tissues. (A) Gene Ontology (GO) enrichment analysis. (B) Kyoto Encyclopedia of Genes and Genomes (KEGG) pathway analysis. Both GO and KEGG analyses were performed on the Metascape server (https://metascape.org/) with the thresholds of minimum overlap = 3, p value cutoff = 0.01, and minimum enrichment = 1.5. [file 4952185.f1.pdf]
